# VIRESCENT-ALBINO LEAF 1 regulates leaf colour development and cell division in rice

**DOI:** 10.1093/jxb/ery250

**Published:** 2018-08-08

**Authors:** Ting Zhang, Ping Feng, Yunfeng Li, Peng Yu, Guoling Yu, Xianchun Sang, Yinghua Ling, Xiaoqin Zeng, Yidan Li, Junyang Huang, Tianquan Zhang, Fangming Zhao, Nan Wang, Changwei Zhang, Zhenglin Yang, Renhong Wu, Guanghua He

**Affiliations:** Rice Research Institute, Key Laboratory of Application and Safety Control of Genetically Modified Crops, Academy of Agricultural Sciences, Southwest University, Chongqing, China

**Keywords:** Cell division, chloroplast development, *de novo* purine biosynthesis, phosphoribosylamine-glycine ligase, pigment metabolism, rice

## Abstract

The *de novo* synthesis of purine nucleotides is crucial to all living organisms, but limited information is available for plants. In this study, we isolated a *virescent-albino leaf 1* (*val1*) mutant of rice (*Oryza sativa*) that produces dynamic green-revertible albino and narrow-leaf phenotypes. In albino leaves, chloroplast development was defective, pigment contents were reduced, and cell division was impaired compared with the wild-type. Map-based cloning revealed that *VAL1* encodes a phosphoribosylamine-glycine ligase (PurD), the second enzyme in the *de novo* purine biosynthesis pathway. Subcellular localization analysis demonstrated that VAL1 was localized in the chloroplast. Our results demonstrate that *VAL1* is a crucial enzyme in the *de novo* purine biosynthesis pathway and is involved in regulating chloroplast development, chlorophyll metabolism, and cell division during leaf development in rice.

## Teaser

The second enzyme of the *de novo* purine biosynthesis pathway, PurD, is involved in leaf-colour development and the cell division process in rice.

## Introduction

Chloroplasts are semi-autonomous organelles responsible for the conversion of energy from sunlight, and for the synthesis of organic molecules by means of photosynthesis in higher plants. Chlorophyll is the primary photosynthetic pigment, and functions in the absorption and transformation of light energy into energy-storage molecules, carbohydrates, and oxygen; it is thus fundamental to the survival and reproduction of all living organisms (Fromme *et al.*, 2003). In higher plants, defective chloroplast development and chlorophyll metabolism lead to changes in chlorophyll content and leaf colour.

Leaf-colour mutants are not only useful as markers in hybrid breeding, but can also be used to study development-related processes such as photosynthesis, chlorophyll metabolism, and chloroplast development, and thus have considerable potential for research and practical application (Du *et al.*, 2009). In higher plants, variation in leaf colour is conspicuous, and in rice its diverse phenotypes include virescent, albino, stripe, chlorina, stay-green, zebra, and yellow variegated forms (Yoo *et al.*, 2009). A substantial number of leaf-colour genes have been mapped, but only a few have been cloned. The two *CAO* homologous genes in rice, *OsCAO1* and *OsCAO2*, encode a chlorophyll *a* oxygenase, which catalyses the conversion of chlorophyll *a* to chlorophyll *b*. Mutation of *OsCAO1* causes a pale-green leaf phenotype (Lee *et al.*, 2005; Yang *et al.*, 2016). *OsChlD*, *OsChlH*, and *OsChlI* encode Mg-chelatase subunits that catalyse the chelation of Mg^2+^ into protoporphyrin IX. Mutations of these three genes all cause a yellow-green leaf phenotype to different degrees of severity (Jung *et al.*, 2003; Deng *et al.*, 2014; Zhang *et al.*, 2015). *YELLOW-GREEN LEAF 1* (*YGL1*) encodes a chlorophyll synthase that catalyses the esterification of a chlorophyllide to complete the final step of chlorophyll biosynthesis in rice. The mutant *ygl1* displays yellow-green leaves in young plants owing to decreased chlorophyll accumulation (Wu *et al.*, 2007). The above-mentioned genes all affect the development of leaf pigmentation by regulating the synthesis and degradation of chlorophyll. Defective chloroplast development can also lead to the formation of leaf-colour mutants. For example, *VIRESCENT 1* (*V1*) encodes a chloroplast protein that regulates chloroplast RNA metabolism and promotes the establishment of the plastid genetic system in rice. *VIRESCENT 2* (*V2*) encodes a novel type of guanylate kinase and is essential for chloroplast development in rice. The *v1* and *v2* mutants each develop chlorotic leaves at restrictive temperatures (Sugimoto *et al.*, 2007). *VIRESCENT 3* (*V3*) and *STRIPE 1* (*St1*) respectively encode the large and small subunits of ribonucleotide reductase, which is required for chloroplast biogenesis in rice. The *v3* and *st1* mutants show chlorotic leaves in a growth stage-dependent manner under field conditions (Yoo *et al.*, 2009). *OsPPR1* encodes a novel pentatricopeptide repeat (PPR) protein, which is essential for chloroplast biogenesis. Antisense transgenic plants display typical chlorophyll-deficient mutant phenotypes of albinism and lethality (Gothandam *et al.*, 2005). Thus, mutations in genes involved in chlorophyll metabolism and chloroplast development result in abnormal leaf-colour phenotypes.

The *de novo* synthesis of the purine nucleotide is crucial for all living organisms. The synthetic pathway is composed of 11 enzymatic steps, leading to the formation of inosinate, and provides the adenine and guanine purines necessary for almost all biochemical processes, including DNA and RNA metabolism, the biosynthetic intermediates ATP and GTP, co-enzymes, and cytokinins, as well as being a vital metabolic regulator in cell signalling (Smith and Atkins, 2002). Although purine plays an important role in plant growth and development, limited information on the synthetic pathway in plants is available. The Arabidopsis *PURINE BIOSYNTHESIS 4* (*PUR4*) gene encodes a formylglycinamidine ribonucleotide synthase, which is the fourth enzyme in the *de novo* purine biosynthesis pathway. The *pur4* mutation is lethal to the male gametophyte and homozygous *pur4* plants cannot be obtained, which indicates that *de novo* purine synthesis is specifically necessary for pollen development (Berthomé *et al.*, 2008). *CIA1* encodes a Gln phosphoribosyl pyrophosphate amidotransferase 2 (ATase2), which is one of the three ATase isozymes responsible for the first committed step of *de novo* purine biosynthesis. The loss-of-function mutant in Arabidopsis and *ATase2*-RNAi transgenic tobacco (*Nicotiana tabacum*) plants show normal green cotyledons but small and albino/pale-green mosaic leaves and reduced chloroplast protein import, which indicates that *de novo* purine biosynthesis is important for chloroplast development. In addition, *de novo* synthesis of purine plays a crucial role in cell division (Hung *et al.*, 2004). *PHOSPHORIBOSYLAMINE-GLYCINE LIGASE* (*PurD*) is the second enzyme in the *de novo* purine biosynthesis pathway, and catalyses the conversion of 5′-phosphoribosylamine (PRA) to 5′-phosphoribosyl-l-glycinamide (GAR) (Smith and Atkins, 2002). In recent years, the biological function of *PurD* in mycelial growth and the virulence of bacterial strains has been extensively studied. For example, strains of *Gibberella zeae* (Kim *et al.*, 2007), *Xanthomonas oryzae* pv. *oryzae* (Park *et al.*, 2007), and *Brucella abortus* (Truong *et al.*, 2015) harbouring mutations in *PurD* show reduced virulence against plants, which indicates that *purD* plays a vital role in the growth and virulence of bacterial strains. However, investigation of the biological functioning of *purD* in plants is incomplete. Previous studies have shown that *purD* is a single-copy and mono-functional enzyme in Arabidopsis, and the gene shows a high degree of conserved homology to *purD* in prokaryotic and eukaryotic species (Schnorr *et al.*, 1994). However, studies on the physiological characteristics and biological function of *purD* in plants are still required.

In this study, we isolated a *virescent-albino leaf 1* (*val1*) mutant, which exhibits a chlorotic and narrow-leaf phenotype, decreased quantities of chlorophyll and carotenoids over the entire growth period, and abnormal chloroplast development on the albino side of the leaf. Map-based cloning revealed that *VAL1* encodes a phosphoribosylamine-glycine ligase, the enzyme involved in the second step of the *de novo* purine biosynthesis pathway. Subcellular localization analysis demonstrated that VAL1 was localized in chloroplasts. VAL1 plays a crucial role in the expression of genes associated with chloroplast development and pigment metabolism. This is the first report to implicate involvement of the *de novo* purine biosynthesis pathway in the development of leaf pigmentation and in cell division, and provides novel insights into the mechanisms underlying chloroplast development, pigment accumulation, and cell division in rice.

## Material and methods

### Plant material

The rice (*Oryza sativa*) *virescent-albino leaf 1* (*val1*) mutant was derived from an ethylmethane sulfonate-treated population of rice ‘Jinhui 10’, which was used as the wild-type (WT) strain for phenotypic observations. The plants were grown in an experimental field at the Southwest University Rice Research Institute (Chongqing, China).

### Physiological analysis

Photosynthetic pigments were extracted from ~0.1 g of newly developed mature leaves from the WT, *val1*, and transgenic plants in 25 ml extraction buffer (ethanol:acetone, 1:1; v/v) for 24 h at room temperature in the dark. The concentrations of chlorophyll *a* and *b*, and carotenoids were determined using a UV-1800PC spectrophotometer (Mapada Co. Ltd, China) at 663, 645, and 470 nm, respectively, and calculated according to the method of Lichtenthaler (1987). The first leaf from the top of the WT, *val1*, and transgenic plants was used to measure photosynthetic rate, stomatal conductance, intercellular CO_2_ concentration, and transpiration rate using a LI-6400 portable photosynthesis meter (LiCor, Lincoln, NE, USA) according to the manufacturer’s instructions. Data presented are the means (±SD) of three biological repeats.

### Transmission electron microscopy

Leaves of the WT, *val1*, and transgenic plants at the tiller stage were collected and cut into 1-mm^2^ squares, fixed in primary fixative solution (3.5% glutaraldehyde), and then post-fixed for 2 h in 1% osmium tetroxide after washing with 0.1 mol l^−1^ phosphate buffer saline. Tissues were stained with uranyl acetate, dehydrated in ethanol, and embedded in Spurr’s medium prior to thin-sectioning. Samples were stained again and examined using a H-7500 TEM (Hitachi, Japan) as described previously (Dong *et al.*, 2013).

### Paraffin-sectioning and histological analysis

Leaves were collected at the tiller stage and fixed in 50% ethanol, 0.9 M glacial acetic acid, and 3.7% formaldehyde for 12 h at 4 °C. The fixed samples were dehydrated with a graded series of ethanol, infiltrated with xylene, and embedded in paraffin (Sigma). A rotary microtome (RM2245; Leica, Hamburg, Germany) was used to cut 8-μm-thick sections, which were transferred onto poly-L-lysine-coated glass slides, deparaffinized in xylene, and dehydrated through an ethanol series (Ren *et al.*, 2018). Light microscopy was performed using an Eclipse E600 microscope (Nikon, Tokyo, Japan).

### Fine-mapping of *VAL1*

The *val1* mutant was crossed with ‘Xinong1A’ (bred by the Southwest University Rice Research Institute) and 2885 F_2_ plants exhibiting the mutational phenotype were selected for high-resolution mapping. Gene fine-mapping was conducted using simple sequence repeat markers obtained from the publicly available rice databases Gramene (http://www.gramene.org) and the Rice Genomic Research Program (http://rgp.dna.affrc.go.jp/E/publicdata/caps/index.html). Insertion/deletion markers were developed from comparisons of genomic sequences from ‘Xinong1A’ and ‘Jinhui 10’ in our laboratory. The sequences of primers used in the fine-mapping and candidate gene analysis are listed in Supplementary Table S1.

### Vector construction for genetic complementation, RNAi, and over-expression

For the complementation test, a 7199-bp genomic fragment was cloned into the binary vector pCAMBIA1301. The resulting recombinant plasmids were introduced into *val1* using the *Agrobacterium tumefaciens*-mediated transformation method as described previously (Zhang *et al.*, 2017). To generate a construct for RNAi, a 328-bp fragment of *VAL1* complementary DNA was amplified and inserted into the vector pTCK303 to obtain the intermediate vector. For overexpression of *VAL1*, the 1580-bp full-length coding sequence of *VAL1* was amplified from ‘Jinhui 10’ cDNA and cloned into vector pTCK303 to generate the intermediate vector. *VAL1* was driven by the rice ubiquitin promoter. The RNAi and overexpression recombinant plasmids were transformed into ‘Jinhui 10’ plants using the *A. tumefaciens*-mediated transformation method (Zhang *et al.*, 2017). The primer sequences are listed in Supplementary Table S1.

### RNA extraction and quantitative real-time PCR analysis

Total rice RNA was extracted from the roots, culms, leaf sheaths, leaves, and young panicles using the RNAprep Pure Plant Kit (Tiangen Co. Ltd, China). The first-strand cDNA was synthesized using the SuperScript^®^ III Reverse Transcriptase Kit (Invitrogen). qRT-PCR analysis was performed using Novostar-SYBR Supermix (Novoprotein, Shanghai, China) in an ABI Biosystems 7500 Real-Time PCR System. At least three replicates were performed and the mean expression level was calculated. Genes used for qRT-PCR analysis were as previously described (Wang *et al.*, 2017). The primer sequences are listed in Supplementary Table S1.

### Multiple sequence alignment and phylogenetic tree construction

Protein sequences were acquired using the BLAST tool in the PHYTOZOME portal, with a 10^−5^ EXPECT value threshold (http://phytozome.jgi.doe.gov/pz/portal.html#!search? show=BLAST). A phylogenetic tree was constructed using MEGA 5.0 with the maximum-likelihood method based on the Jones–Taylor–Thornton matrix-based model with the lowest Bayesian information criterion scores (Tamura *et al.*, 2011).

### Nucleotide and cytokinin measurements

Samples of the WT and *val1* mutant seedlings (0.1 g) were frozen in liquid nitrogen and homogenized. Nucleotides were extracted using the trichloroacetic acid (TCA) method as described previously (Hajirezaei *et al.*, 2003). The nucleotide and cytokinin contents in the TCA extracts were measured using a HPLC system (Kontron, Eching, Germany) fitted with a Partisil-SAX anion-exchange column, and detected by absorption at OD_254_. Identification and quantification was completed by comparison with standards, as described previously (Hajirezaei *et al.*, 2003).

### Nucleotide treatment *in vitro*

Wild-type and *val1* seeds were surface-sterilized after husking and grown in half-strength Murashige and Skoog medium (pH 5.8) containing different concentrations (0, 1, 5, and 10 mM) of adenine nucleotide (AMP), guanine nucleotide (GMP), or AMP and GMP. The seeds were incubated at 28 °C under a 16/8 h light/dark photoperiod. Continual observation of seedling phenotypes was undertaken for 7–10 d.

### Subcellular localization

The full-length coding region of *VAL1* was amplified and cloned into the expression cassette 35S-*GFP*-NOS (pA7) in order to generate the *VAL1-GFP* (green fluorescent protein) fusion vector. Both pA7-*VAL1-GFP* and empty pA7-*GFP* plasmids were transformed into rice protoplasts as described previously (Ma *et al.*, 2017). After overnight incubation at 28 °C, GFP fluorescence was observed using a confocal laser-scanning microscopy (Olympus FLUOVIEW FV1000; Japan). The primer sequences are listed in Supplementary Table S1.

### *In situ* hybridization

For the *VAL1* and *His4* probes, a gene-specific cDNA was amplified and labelled using a DIG RNA Labelling Kit (Roche). Pretreatment of sections, hybridization, and immunological detection were performed as described previously (Zhang *et al.*, 2017). The primer sequences are listed in Supplementary Table S1.

### Western blot analysis

Total proteins from 10-d-old WT and *val1* seedlings were isolated using a Plant Total Protein Extraction Kit (Sangon Biotech). An equal amount of protein was separated on 10% SDS-polyacrylamide gels. The proteins were transferred to iBlot 2 PVDF Regular Stacks using the iBlot 2 Gel Transfer Device (Invitrogen), immunoblotted with various primary antibodies (Agrisera and Beijing Protein Innovation Company) and AP-conjugated Affinipure Goat Anti-Rabbit IgG (Jackson ImmunoResearch) according to the recommended dilution ratios in the iBind Cards using the iBind Western Device (Invitrogen), and detected using the NBT/BCIP substrate (Sangon Biotech).

### Transcriptome analysis

Seedlings of the WT and *val1* mutant were used for RNA extraction and transcriptome sequencing with two biological replicates. The purity and concentration of RNA were measured using a NanoPhotometer spectrophotometer (Implen, Westlake Village, CA, USA) and a Qubit^®^ RNA Assay Kit in a Qubit^®^ 2.0 Fluorometer (Life Technologies, Carlsbad, CA, USA). Library construction and RNA sequencing (RNA-seq) were conducted by Omicgene Biological Technology (Wuhan, China) on a HiSeq X Ten platform (Illumina, San Diego, CA, USA). RNA-seq short reads were aligned to the *indica* rice genome with HISAT2 (https://ccb.jhu.edu/software/hisat2/index.shtml), and the expression level of each gene was computed using StringTie (https://ccb.jhu.edu/software/stringtie/). The expression-level data were analysed using the gene set enrichment analysis (GSEA) software (version 2.07) to identify functionally related groups of genes (gene sets) with statistically significant enrichment (Subramanian *et al.*, 2005). The gene set files used in GSEA were constructed in accordance with the genome annotation (http://www.mbkbase.org/R498). The significance of differentially expressed genes (DGEs) was determined using *q*-values <0.005 and |log_2_ (fold-change)| >1. Gene Ontology (http://www.geneontology.org/) analyses were performed by referring to GOseq (http://dx.doi.org/10.18129/B9.bioc.goseq).

### Flow cytometric analysis

For nuclear staining, the shoot apex from rice seedlings was cut into small pieces in 400 µl Partec CyStain UV Precise P Lysis solution (Sysmex Partec) on ice, and then filtered with a 30-µm filter to remove cellular debris. After staining with Partec CyStain UV Precise P Staining solution (including DAPI) (Sysmex Partec), the nuclei were analysed using CyFlow Space (Sysmex Partec). A total of 30 000 events were recorded, and the data were analysed using the FCS Express software (https://www.denovosoftware.com/).

## Results

### The *val1* mutation confers a green-revertible albino and narrow-leaf phenotype

**Fig. 1.**
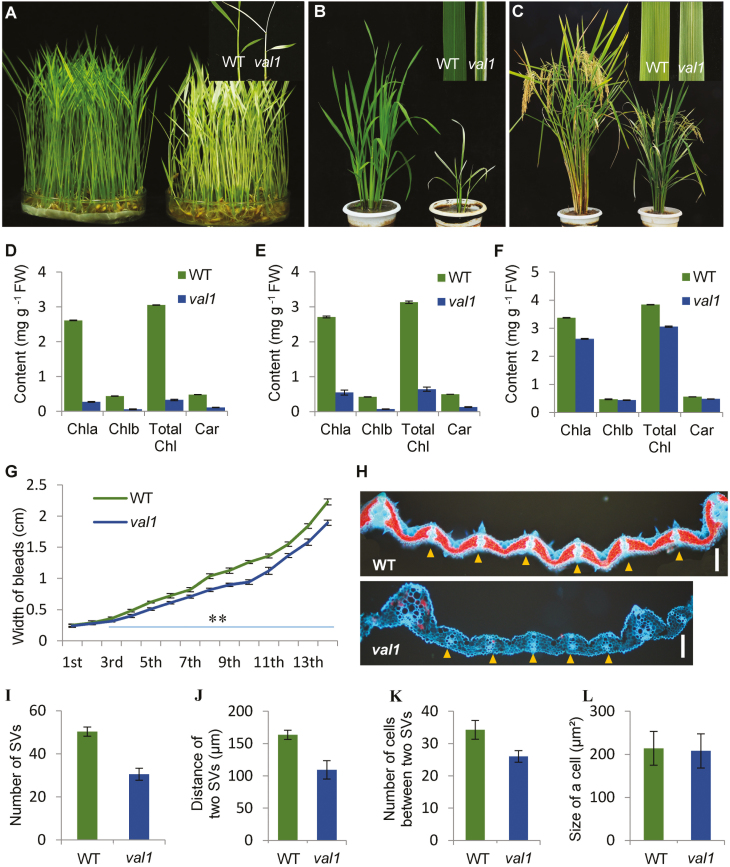
Phenotypic characteristics of the wild-type (WT) and the *val1* mutant. (A–C) Phenotypes of the WT and *val1* at the seedling stage (A), tiller stage (B), and heading stage (C). Close-up images of the leaves of the WT and *val1* are shown at top-right. (D–F) Chlorophyll content of the leaves of the WT and *val1* at the seedling stage (D), tiller stage (E), and heading stage (F). (G) Leaf blade widths of the WT and *val1* plants for the 1st–14th leaves produced across the total growth period. (H) Transverse sections of the seventh leaf blade of the WT and *val1*. Yellow triangles indicate small vascular bundles. Scale bars are 100 µm. (I–L) Comparisons of the number of small vascular bundles (SV) (I), the distance between the small vascular bundles (J), and the number of cells (K) and the size of cells (L) between the small vascular bundles in the WT and *val1*. Data are means (±SD) of three biological replicates.

In the wild type (WT), the leaves of the plant appeared green throughout the growth period except in the later mature stage. In contrast, in the *val1* mutant, new albino leaves were developed at the early seedling stage (Fig. 1A). The leaves subsequently turned green with an albino margin and displayed a mixed phenotype in which green and albino leaves were present from the later seedling stage to the tiller stage (Fig. 1B). During the heading stage, the leaves were almost gray-green in colour, and only a small number of leaves showed marginal albinism (Fig. 1C). Thus, the older the leaves, the smaller the area of albinism (Fig. 1A–C). These observations demonstrated that the phenotype of *val1* was displayed in a growth stage-dependent manner. In addition, in accordance with the albino leaf phenotype of the *val1* mutant, the leaf chlorophyll content was significantly reduced in the mutant compared with the WT at the seedling and tiller stages (Fig. 1D, E), and slightly reduced at the mature stage (Fig. 1F). Taken together, these observations over the full life cycle demonstrated that *val1* was a dynamic green-revertible albino mutant. We also examined the expression of the phenotypes and the leaf pigment contents in relation to temperature. The *val1* mutant always displayed albino leaves with lower pigment contents than those of the WT at temperatures of 20 °C (C20), 30 °C (C30), and under an alternate light/dark cycle (12 h light at 30 °C/12 h dark at 20 °C; L30 °C/D20 °C) (Supplementary Fig. S1). These results indicated that the albino-leaf phenotype of *val1* in the early seedling stage was not affected by temperature, and we therefore determined that *val1* was a temperature-insensitive, green-revertible albino mutant.

The leaf width in *val1* was reduced significantly compared with the WT, and the narrow-leaf phenotype was increasingly pronounced over the course of plant development (Fig. 1A–C, G). To examine the basis for the narrow-leaf phenotype, histological analysis of transverse sections of the leaf blade showed that, compared with the WT, the number of small vascular bundles was reduced by 39.3% (Fig. 1H, I), and the distance between the small vascular bundles was reduced by 33.2% (Fig. 1H, J). The number of cells between the small vascular bundles was reduced by 24.1% (Fig. 1H, K), but the size of the cells was similar to that of the WT (Fig. 1H, L). These results indicated that the reduced leaf width may have been caused by the reduced number of vascular bundles and the reduced number of cells between the small vascular bundles. In addition, *val1* plants also displayed dwarfism and smaller spikelets compared with the WT (Fig. 1A–C).

### Molecular cloning and identification of *VAL1*

**Fig. 2.**
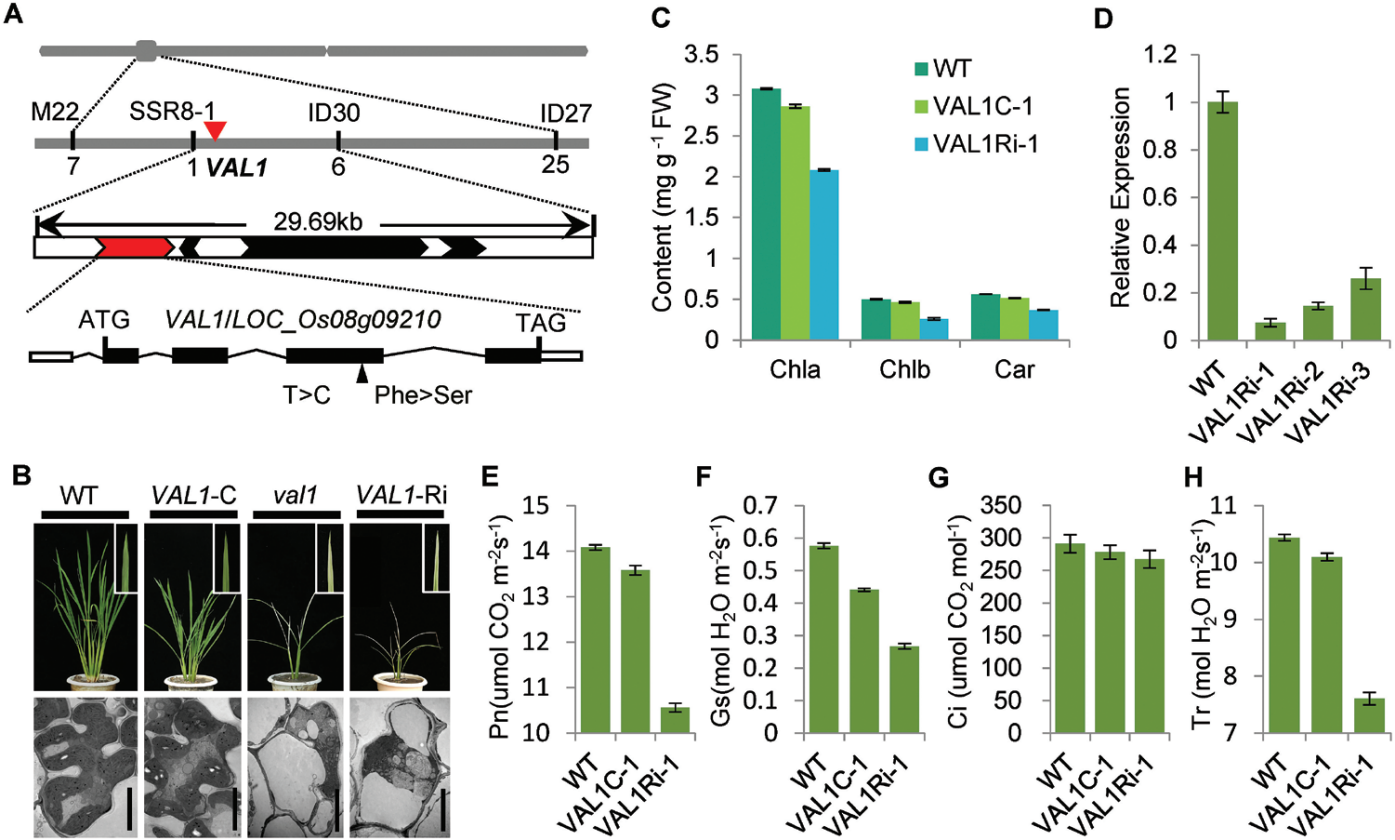
Molecular identity of *VAL1*. (A) Map-based cloning of the *VAL1* gene. (B) Phenotypes of the wild-type (WT), complemented (-C), *val1*, and *VAL1*-RNAi plants. Scale bars are 5 µm. (C) Chlorophyll contents of the leaves of the WT, complemented, and *VAL1*-RNAi plants at the tiller stage. (D) Expression analysis of *VAL1* in the leaves of the WT and the three RNAi lines by qRT-PCR. (E–H) Photosynthetic rate (E), stomatal conductance (F), intercellular CO_2_ concentration (G), and transpiration rate (H) of the WT, complemented, and *VAL1*-RNAi plants at the heading stage. Data are means (±SD) of three biological replicates.

The *VAL1* gene was previously mapped to a region of about 171 kb on chromosome 8 (Ma, 2014). In the current study, the location of *VAL1* was narrowed to within a physical distance of 29.69 kb between the simple sequence repeat marker SSR8-1 and the insertion/deletion marker ID30. This interval includes four annotated genes (http://www.gramene.org/). Sequencing analysis identified a single-nucleotide substitution from T to C within the *LOC_Os08g09210* gene, causing an amino acid mutation of Phe-358 to Ser-358 in the *val1* mutant (Fig. 2A). To confirm whether the mutation of *LOC_Os08g09210* resulted in the mutant phenotype, we performed a complementation experiment by transforming a 7198-bp WT DNA fragment containing *LOC_Os08g09210* into the *val1* mutant. The mutant phenotype was completely rescued in the transgenic plants (Fig. 2B). The contents of chlorophyll and carotenoids and the photosynthetic rate in the transgenic plants were almost identical to those of the WT (Fig. 2C, E–H). In addition, the chloroplast ultrastructure as observed by TEM was similar to that of the WT, displaying well-developed lamellar structures and equipped with normal thylakoid membranes and stacked grana (Fig. 2B). We performed RNAi to silence *VAL1* in the WT plants. In the transgenic plants, the quantity of *VAL1* transcripts was greatly reduced (Fig. 2D), and a dynamic green phenotype similar to that of the *val1* mutant was observed (Fig. 2B). The contents of chlorophyll and carotenoids and the photosynthetic rate in the transgenic plants were significantly reduced compared with the WT (Fig. 2C, E–H). Observation by TEM showed that the mesophyll cells were almost empty and lacked obvious intact organelles, and the chloroplasts were completely degraded, similar to those of *val1* (Fig. 2B). Taken together, these results confirmed that *LOC_Os08g09210* corresponded to the *VAL1* gene.

### Expression pattern of VAL1 and subcellular localisation of the VAL1 protein

**Fig. 3.**
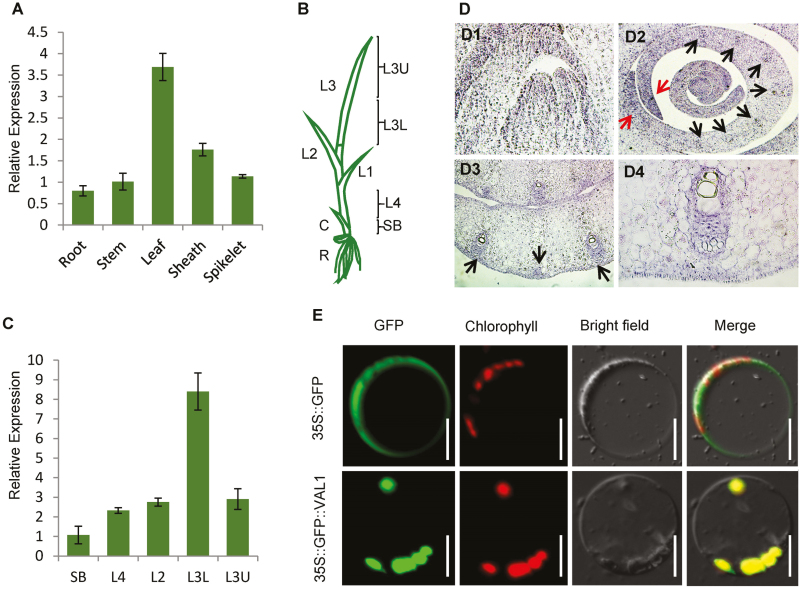
Expression patterns of *VAL1* and subcellular localization of the VAL1 protein. (A) Expression pattern of *VAL1* in different tissues as indicated by real-time PCR. (B, C) Expression patterns of *VAL1* in the leaf as indicated by real-time PCR. (D) Expression patterns of *VAL1* by *in situ* hybridization. Black arrows indicate the procambium cells and vascular bundles. Red arrows indicate the margin of the P3 primordium. (E) Analysis of the subcellular localization of the VAL1 protein in rice protoplasts. Scale bars are 50 µm. SB, shoot base; L4, fourth leaf; L2, second leaf; L3L, basal half of the third leaf; L3U, upper half of the third leaf. Data are means (±SD) of three biological replicates.

To determine the expression pattern of *VAL1*, quantitative reverse transcription-PCR (qRT-PCR) analysis was performed. *VAL1* was expressed in a variety of tissues, including the roots, stems, leaves, leaf sheaths, and panicles, with higher levels in the leaves (with higher chlorophyll content) than in the other tissues examined (Fig. 3A). Previous studies have shown that the leaves of rice emerge from the sheath in an ordered manner. Generally, when the third leaf has fully emerged from the sheath, the shoot includes the fourth to seventh immature leaves. The leaf cells in the upper half of the third leaf (L3U) and basal (lower) half of the third leaf (L3L) contain mature chloroplasts, whereas those in the shoot base (SB) and fourth leaf above the shoot base (L4) contain proplastids and early-developing immature chloroplasts (Fig. 3B; Yoo *et al.*, 2009). In our present study, qRT-PCR analysis showed that *VAL1* was universally expressed in the SB, L4, L2, L3L, and L3U samples, with the highest levels detected in L3L (Fig. 3C).

For a more detailed analysis of the expression pattern of *VAL1*, transverse and longitudinal sections of the shoot apical meristem (SAM) were examined for *VAL1* signals by *in situ* hybridization (Fig. 3D). First, strong signals were observed in the SAM (Fig. 3D1). Next, scattered strong signals were detected in the P2 primordia (Fig. 3D2). In the P3 primordia, *VAL1* signals were concentrated at the margin of the P3 primordium and the procambium cells (Fig. 3D2), which may predict the sites of differentiation of procambium cells from fundamental cells. In the P4 primordia, *VAL1* signals were primarily restricted to the vascular bundles, with no preference for the xylem or phloem (Fig. 3D3, D4). These results suggested that VAL1 may be involved in leaf development.

To examine the subcellular localization of the VAL1 protein, constructs producing the GFP and the GFP-VAL1 ORF fusion protein were transiently expressed in rice. In cells that expressed GFP alone, green fluorescence was detected consistently throughout the cell except in the vacuole in protoplasts, and the GFP-VAL1 ORF fusion protein was localized in the chloroplast (Fig. 3E). These results indicated that VAL1 was localized in the chloroplast.

### *VAL1* encodes a phosphoribosylamine-glycine ligase

A blastp analysis was performed on the NCBI protein database using the VAL1 protein sequence. *VAL1* encodes a phosphoribosylamine-glycine ligase (PurD), which belongs to the ATP-grasp superfamily. Alignment of amino acid sequences of VAL1 and other phosphoribosylamine-glycine ligase family members revealed that the sequences showed a high degree of homology. The protein structure was analysed using the PSIPRED protein prediction database (http://bioinf.cs.ucl.ac.uk/psipred/). VAL1 was predicted to include three conserved domains, designated GARS_N, GARS_A, and GRAS_C, of which the GARS_A domain contained an ATP-binding site. In addition, it was predicted by ChloroP (http://www.cbs.dtu.dk/services/ChloroP/) that VAL1 contained a chloroplast transit peptide at the N-terminus (amino acid residues 1–68; Supplementary Fig. S2).

**Fig. 4.**
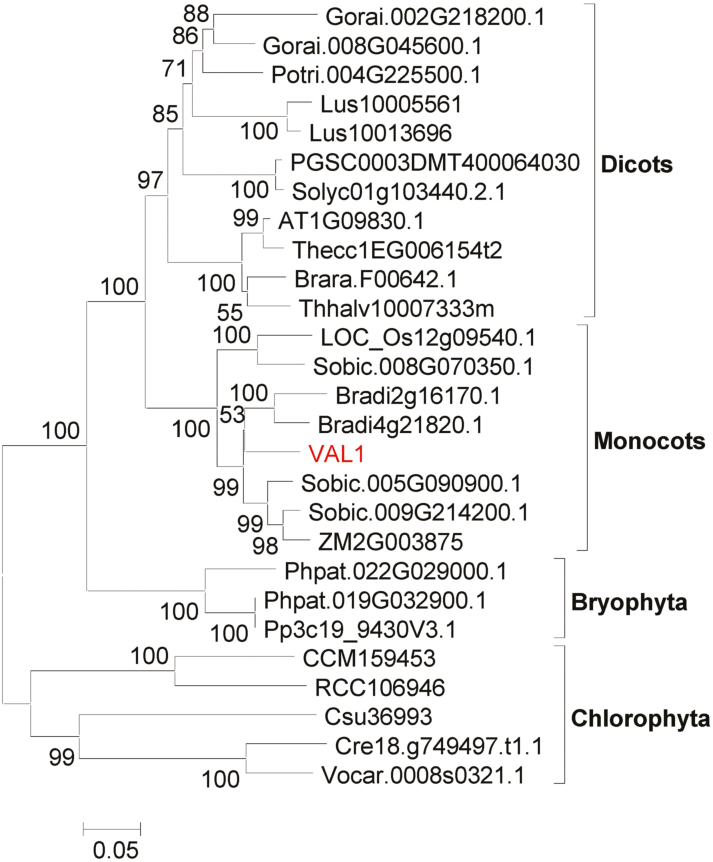
Phylogenetic tree for the VAL1 protein. The tree was constructed using the maximum-likelihood method based on the Jones–Taylor–Thornton matrix-based model. Bootstrap values calculated from 500 replicates are given at the branch nodes. The VAL1 protein is highlighted in red.

The phylogenetic tree indicated that VAL1 is widely present in many photosynthetic organisms (Fig. 4), including lower aquatic algae, terrestrial ferns, gymnosperms, and angiosperms, and it showed a high degree of homology, which suggests that VAL1 in rice may be descended from VAL1 of algae. In addition, the rice genome contains a *VAL1* homolog designated *LOC_Os12g09540*, which is also predicted to encode a phosphoribosylamine-glycine ligase. *LOC_Os12g09540* was also expressed in leaves, with the highest levels detected in L3U (Supplementary Fig. S3A, B). However, LOC_Os12g09540 and VAL1 do not belong to the same evolutionary lineage, which indicates a certain degree of differentiation in molecular function between the homologs.

### Complementation of the nucleotide *in vitro* partially restores the phenotype

**Fig. 5.**
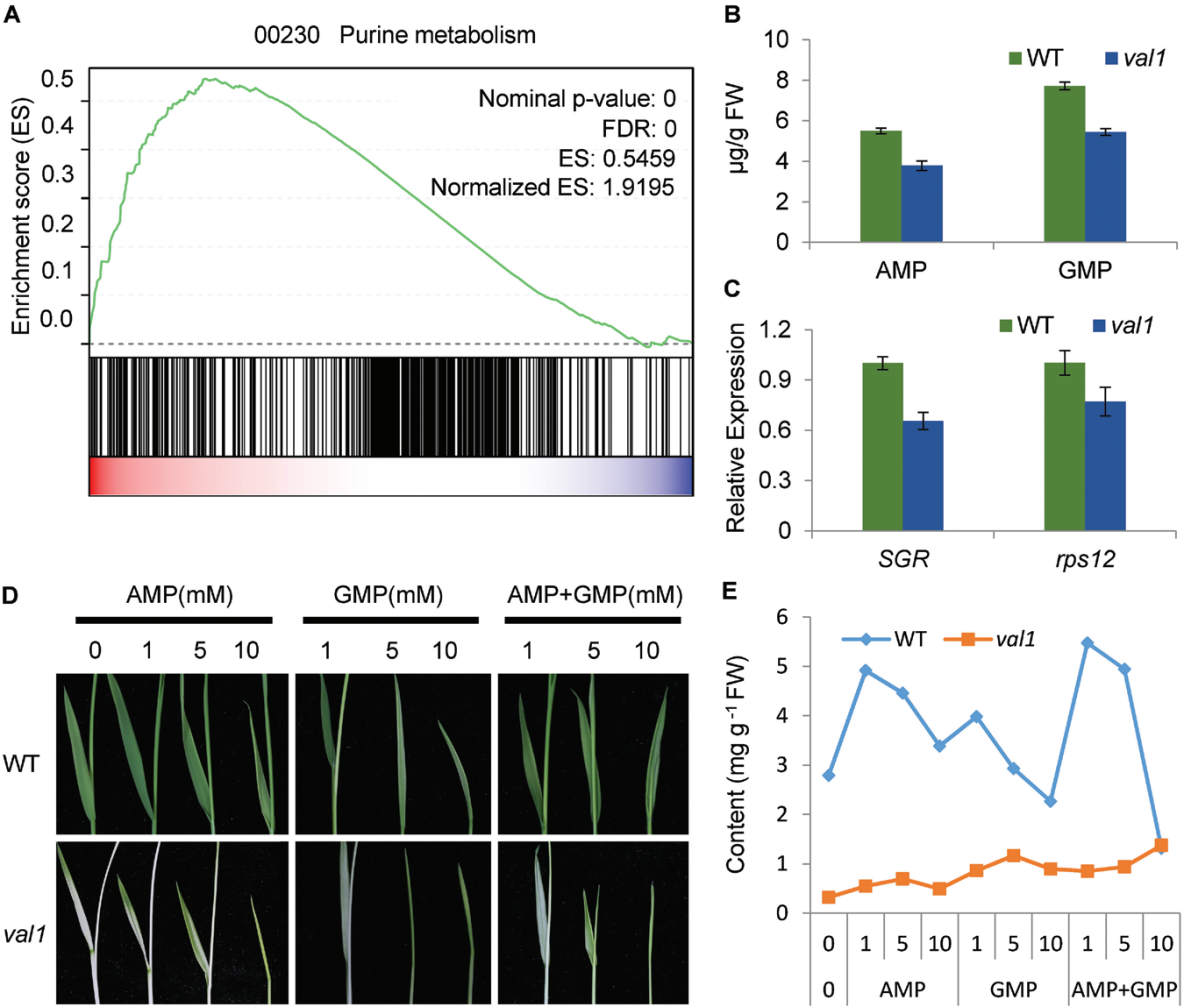
Phosphoribosylamine-glycine ligase (PurD) and *de novo* purine biosynthesis. (A) Gene set enrichment analysis of genes associated with the *de novo* purine biosynthesis pathway. The green line indicates enrichment score calculated by gene set enrichment analysis (GSEA). (B) AMP and GMP contents in the wild-type (WT) and *val1* mutant. (C) Expression of *rps12* and *SGR* in seedlings of the WT and *val1*. (D) Leaf morphology of the WT and *val1* mutant after growth in Murashige and Skoog liquid medium supplemented with various concentrations (0, 1, 5, or 10 mM) of AMP, GMP, or AMP and GMP together for 2 weeks. (E) Chlorophyll content of plants of the WT and *val1* mutant shown in (D). Data are means (±SD) of three biological replicates.

*VAL1* encodes a phosphoribosylamine-glycine ligase, which is the second enzyme in the *de novo* purine biosynthesis pathway (Supplementary Fig. S4A). To confirm its involvement in the pathway, the transcriptomic profiles of seedlings of *val1* and WT grown under normal conditions were characterized by RNA-seq with two biological replicates for each genotype. The Pearson correlation coefficient between the two replicates was greater than 0.95. Gene set enrichment analysis (GSEA) was used to examine the data. This is a statistical approach that allows identification of over-represented gene sets among differentially up- or down-regulated genes of a transcriptomic analysis (Subramanian *et al.*, 2005). There was significant enrichment in the over-representation of genes associated with the *de novo* purine biosynthesis pathway (Fig. 5A, Supplementary Fig. S4B), which indicated that the mutation of *VAL1* caused disruption of *de novo* purine biosynthesis. In addition, the contents of the *de novo* purine biosynthesis products AMP and GMP in v*al1* seedlings were lower than those of the WT (Fig. 5B), which further indicated that VAL1 was involved in the *de novo* purine biosynthesis pathway.

The decreased AMP and GMP contents in the *val1* mutant may influence plastid genome replication and division. We therefore used qRT-PCR to examine the expression of *SGR* and *rps12*, which are single-copy genes in the plant nuclear and plastid genomes, as representatives of the copy numbers of nuclear and chloroplastic genomes, respectively (Sugimoto *et al.*, 2007; Yoo *et al.*, 2009). The expression levels of both *SGR* and *rps12* were reduced in the *val1* mutant (Fig. 5C), which indicated that nuclear and chloroplastic genome replication appeared to be arrested by limited AMP and GMP levels.

Next, we investigated whether the mutant phenotypes could be complemented biochemically by exogenous application of AMP and GMP. Application of AMP, GMP, and AMP and GMP together partially restored the green colour of the *val1* leaf (Fig. 5D). Low concentrations (1 and 5 mM) of AMP, GMP, and AMP and GMP promoted accumulation of chlorophyll in WT leaves, whereas high concentrations (10 mM) repressed chlorophyll accumulation and inhibited plant growth (Fig. 5D). In the *val1* mutant, chlorophyll content was elevated with increasing concentrations of AMP, GMP, and AMP and GMP (Fig. 5E). Thus, the applications partially restored the accumulation of chlorophyll in the *val1* mutant. In addition, low concentrations (10 and 100 nM) of the synthetic cytokinin benzylaminopurine (6-BA) did not rescue the mutant phenotype, and a high concentration of 6-BA (1000 nM) severely inhibited plant growth (Supplementary Fig. S5).

### *VAL1* affects chloroplast development and pigment metabolism

**Fig. 6.**
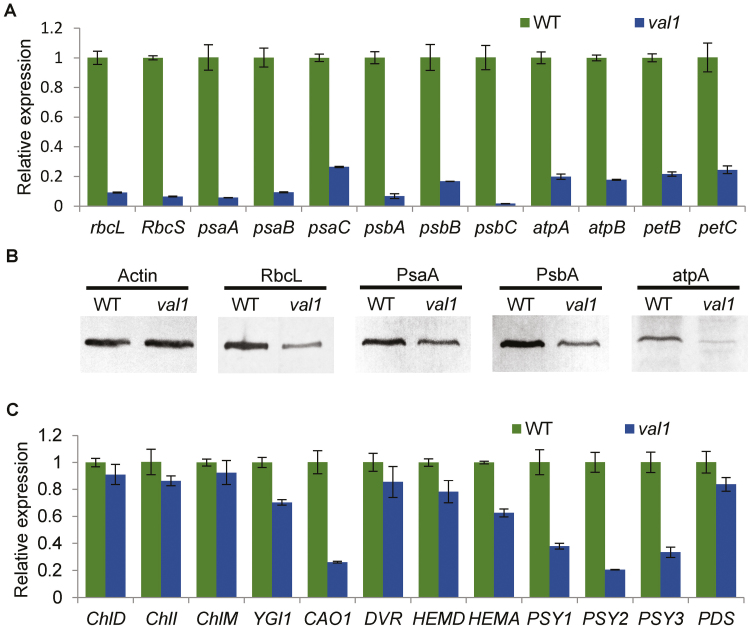
Expression of genes associated with chloroplast development, photosynthesis, and pigment metabolism in the wild-type (WT) and *val1* mutant. (A) Expression analysis of genes associated with chloroplast development and photosynthesis in the WT and *val1*. (B) Western blots of proteins associated with chloroplast development and photosynthesis. Actin was used as an internal control. (C) Expression analysis of genes associated with pigment metabolism in the WT and *val1*. Data are means (±SD) of three biological replicates.

Previous research has indicated that chloroplast development in the albino portion of the leaf in *val1* plants is severely defective (Ma, 2014). Thus, expression analysis of genes associated with chloroplast development and photosynthesis were compared between WT and *val1* plants by qRT-PCR analysis. The transcript levels of *rbcL* and *RbcS* (encoding the large and small subunit of Rubisco, respectively), *psaA*, *psaB*, *psaC*, and *psbA*, *psbB*, *psbC* (encoding two reaction-centre polypeptides of PSI and PSII, respectively), *atpA* and *atpB* (encoding the alpha- and beta-subunits of chloroplast ATP synthase, respectively), *petB* and *petC* (encoding the cytochrome b6 subunit and the Rieske FeS centre of cytochrome b6f complex, respectively) were significantly reduced in the *val1* mutant compared with the WT (Fig. 6A). The protein accumulation levels of RbcL, PsaA, PsbA, and atpA were consistent with the transcript levels (Fig. 6B). Taken together, these results indicated that the *VAL1* mutation affected the expression of genes associated with chloroplast development and photosynthesis, which resulted in defective chloroplast development.

Given that the *val1* mutant displayed reduced chlorophyll and carotenoid contents, the expression levels of pigment metabolism-related genes were compared between WT and *val1* plants by qRT-PCR analysis. The transcript levels of pigment metabolism-related genes were reduced in the *val1* mutant compared with those of the WT to different degrees. For example, the transcript levels of the chlorophyll synthesis pathway gene *CAO* and the carotenoid synthesis pathway genes *PSY1*, *PSY2*, and *PSY3* were all significantly reduced in the *val1* mutant compared with the WT. The other genes involved in the synthesis of chlorophyll and carotenoids, such as *ChlD*, *ChlI*, *ChlM*, *YGL1*, *DVR*, *HEMA*, *HEMD*, and *PDS* were also slightly reduced in the *val1* mutant compared with the WT (Fig. 6C). In the heading stage, the expression of these genes related to chloroplast development, photosynthesis, and pigment metabolism were not significantly reduced in the *val1* mutant compared with the WT (Supplementary Fig. S6A, B).

To obtain further insights into the function of VAL1, the transcriptomic profiles of seedings of *val1* and the WT were characterized. A total of 3683 DEGs between *val1* and WT were identified, including 1813 up- and 1870 down-regulated DEGs. GO enrichment analysis showed that the expression of genes involved in chloroplast development, photosynthesis, and pigment biosynthesis were changed dramatically. For chloroplast development and photosynthesis a total of 34 DEGs were identified, comprising 32 down- and two up-regulated genes; for example, the expression of *rbcL*, *psaG*,and *psbR* showed decreases of 53.6, 74.1, and 82.6%, respectively, in *val1* (Supplementary Table S2). For pigment biosynthesis a total of 25 DEGs were identified, comprising 16 down- and nine up-regulated genes; for example, the expression of *PORA* and *CAO1* showed decreases of 85.9% and 47.5%, respectively, in *val1* (Supplementary Table S3).

### *VAL1* is involved in cell division in leaves

**Fig. 7.**
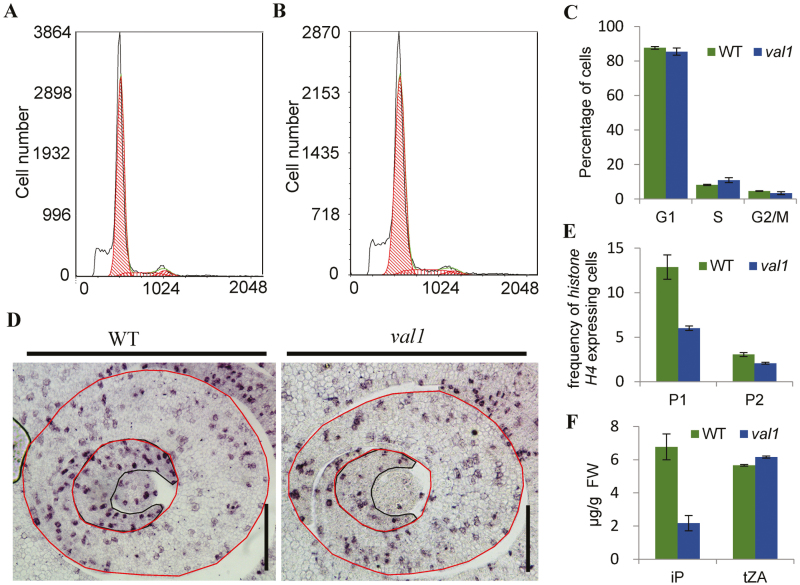
Involvement of VAL1 in cell division in leaves. (A, B) Flow cytometry measurements of nuclei from the shoot apex in 10-d-old wild-type (WT) (A) and *val1* plants (B). (C) Quantification of the DNA profiles of WT and *val1* plants. (D) Expression of *histone H4* in cross-sections of the shoot apical meristems in the WT and *val1* at the tiller stage. Black and red lines represent P1 and P2 primordia, respectively. Scale bars are 100 µm. (E) Frequency of *histone H4*-expressing cells in the leaf primordia indicated by red circles in (D). P1, P1 promordium; P2, P2 promordium. (F) Contents of isopentenyladenine and *trans*-zeatin in the WT and *val1* mutant seedlings. Data are means (±SD) of three biological replicates.

Since the number of vascular bundles and cells between the small vascular bundles was significantly reduced in the narrow leaves of *val1*, we speculated that this may have been caused by impaired cell proliferation of leaf primordia. To text this hypothesis, flow cytometry was used to monitor the amount of DNA in the nuclear suspensions in the shoot apex of WT and *val1* plants. The results revealed a significant increase in the number of cells in the S phase and decreases in the G2/M phases of the cell cycle, implying that the cell cycle was delayed at the S phase (Fig. 7A–C). The expression patterns of *histone H4* were characterized by *in situ* hybridization and it was found to be relatively evenly expressed throughout the P1 leaf primordium in the WT, whereas the frequency of cells that expressed *histone H4* was reduced by 29.2% in the *val1* mutant (Fig. 7D, E). In the P2 leaf primordium, *histone H4* was mainly expressed at the margins of the primordium, and the frequency of cells that expressed *histone H4* was reduced by 27.7% in the *val1* mutant compared with the WT (Fig. 7D, E). These results confirmed that the decreased number of cells was caused by impaired cell proliferation during the early development of primordia. Given that *de novo* purine synthesis leads to the synthesis of cytokinins, we measured the contents of isopentenyladenine (iP) and *trans*-zeatin (tZA) in WT and v*al1* seedlings. The content of iP was reduced by 67.9%, whereas that of tZA was increased slightly in the *val1* mutant compared with the WT (Fig. 7F). Taken together, these results supported the contention that the decreased number of cells was caused by impaired cell proliferation, which may have been due to the reduced content of cytokinins during the early development of leaf primordia in the *val1* mutant.

### Application value of *VAL1* in rice breeding

**Fig. 8.**
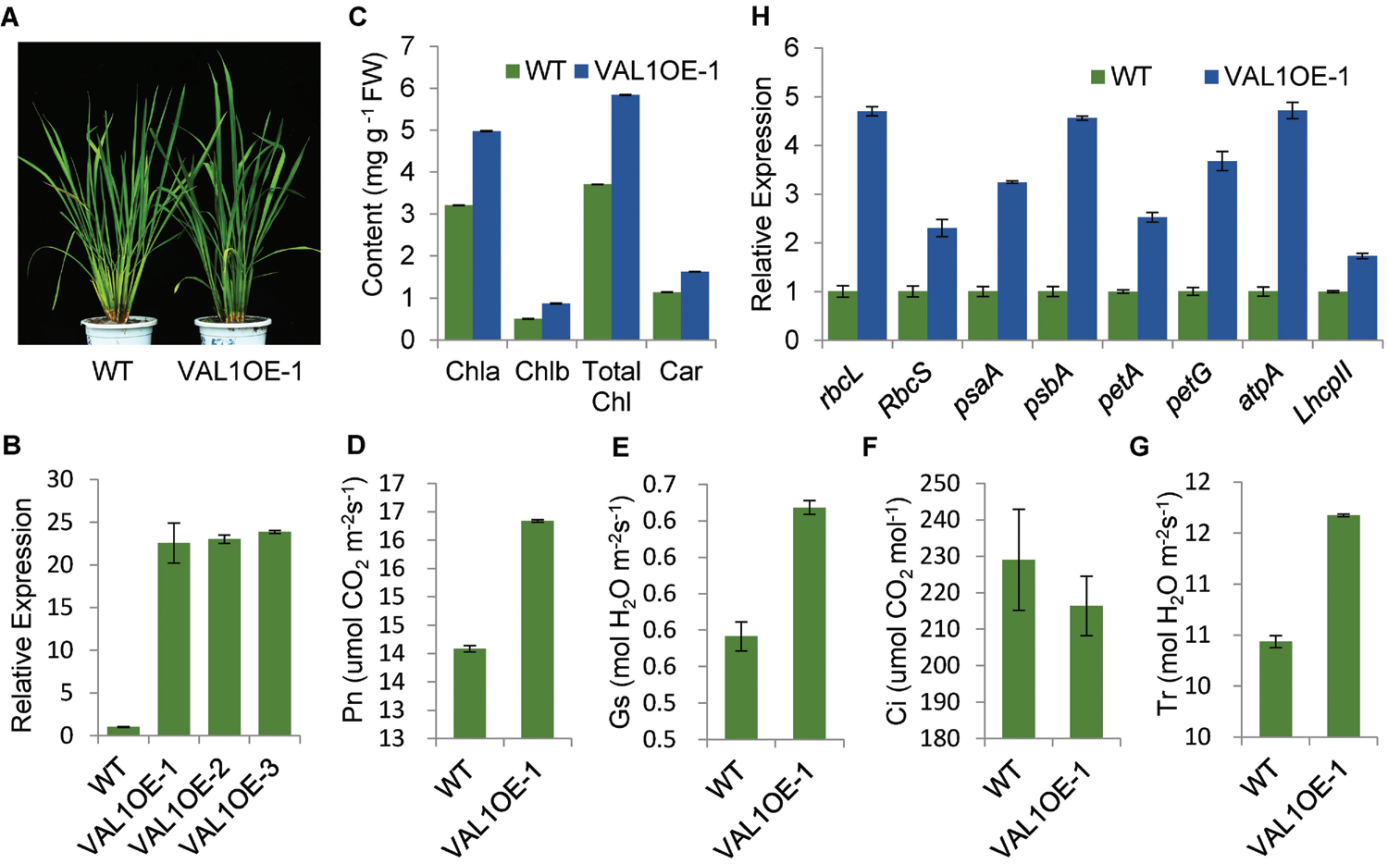
Application value of *VAL1* in rice breeding. (A) Phenotypes of the wild-type (WT) and *VAL1*-overexpressing plants in the tiller stage. (B) Expression analysis of *VAL1* in the leaf of the WT and three *VAL1*-overexpressing lines by real-time PCR. (C) Chlorophyll content of the leaf of the WT and *VAL1*-overexpressing plants in the tiller stage. (D–G) Photosynthetic rate (D), stomatal conductance (E), intercellular CO_2_ concentration (F), and transpiration rate (G) of the WT and *VAL1*-overexpressing plants at the heading stage. (H) Expression analysis of genes associated with photosynthesis in the WT and *VAL1*-overexpressing plants. Data are means (±SD) of three biological replicates.

The shade of the leaf colour in rice is determined by the content of chlorophyll. Chlorophyll is the main pigment of plants for photosynthesis and plays a key role in the absorption, transmission, and conversion of light energy (Fromme *et al.*, 2003). To assess the application value of *VAL1* in rice breeding, we expressed *VAL1* under the control of the ubiquitin promoter in the WT. In the tillering stage, the green leaf colour was slightly darker in transgenic plants compared with the WT (Fig. 8A). The chlorophyll and carotenoid contents were significantly increased (Fig. 8C), and the photosynthetic rate was increased in transgenic plants compared with the WT (Fig. 8 D). The qRT-PCR results showed that the expression level of *VAL1* was significantly increased in transgenic plants compared with the WT (Fig. 8B). The expression levels of photosynthesis-associated genes *rbcL*, *RbcS*, *psaA*, *psbA*, *petA*, *petG*, *atpA*, and *LhcpII*, were significantly increased to different degrees in transgenic plants compared with the WT (Fig. 8H). These results indicated that VAL1 played an important role in regulation of plant photosynthesis by modulating the expression of related genes. Thus, plants with overexpressed *VAL1* showed a higher photosynthetic ability, and could hence they could be utilized in breeding.

## Discussion

In our present study, *VAL1* was shown to encode a phosphoribosylamine-glycine ligase, the second enzyme in the *de novo* purine biosynthesis pathway. Subcellular localization indicated that the VAL1 protein was localized in the chloroplast (Fig. 3). VAL1 is a novel regulator that regulates chloroplast development, pigment metabolism, and cell division, and thereby affects the development of leaf colour and blade width in rice.

Many genes associated with leaf colour have been cloned in rice. Metabolic pathways associated with chlorophyll synthesis and degradation, and chloroplast development have been elucidated, and mutation of genes involved in these metabolic pathways leads to changes in leaf colour. For example, mutations in the genes *OsCHlH* (Jung *et al.*, 2003), *OsCHlD* and *OsCHlI* (Zhang *et al.*, 2015), *OsDVR* (Wang *et al.*, 2010), *OsGluRs* (Liu *et al.*, 2007), *YGL1* (Wu *et al.*, 2007), *OsCAO1* and *OsCAO2* (Lee *et al.*, 2005; Yang *et al.*, 2016), *OsPPR1* (Gothandam *et al.*, 2005), *YSA* (Su *et al.*, 2012), *SGR* (Jiang *et al.*, 2007), *NYC1* (Kusaba *et al.*, 2007), *V1* (Kusumi *et al.*, 2011), *V2* (Sugimoto *et al.*, 2004), and *V3* and *st1* (Yoo *et al.*, 2009) produce different types of leaf-colour mutants. In the *val1* mutant, the expression levels of chlorophyll- and photosynthesis-associated genes were significantly reduced, and chloroplast development was defective (Figs 2D, 6). Thus, similar to the above-mentioned leaf-colour mutants, the green-revertible albino phenotype of the *val1* mutant was caused by disruption of chlorophyll metabolism and chloroplast development. Previous research has indicated that the phenotypes of the majority of green-revertible albino leaf-colour mutants are regulated by temperature and development, or by a combination of the two. For example, the temperature-sensitive seedling-colour mutant *tsc-1* displays a white, light-yellow, and normal green leaf when grown at 23.1 °C, 26.1 °C, and 30.1 °C, respectively (Dong *et al.*, 2001). In contrast, the albino leaf phenotype of the *val1* mutant at the early seedling stage was not affected by temperature, which indicated that *val1* is a temperature-insensitive mutant (Supplementary Fig. S1). Developmentally regulated mutants, such as the *ysa* mutant, develop albino leaves before the three-leaf (L3) stage and gradually turn green and recover to the normal green leaf colour at the six-leaf stage (Su *et al.*, 2012). The temperature-conditional and developmentally-regulated mutants *v3* and *st1* largely exhibit a normal green-leaf phenotype up to the L3 stage and develop chlorotic leaves until the maximum tiller stage, but recover to develop mostly green leaves after heading. At temperatures of 20 °C or 30 °C, these mutants produce chlorotic leaves, whereas under an alternate light/dark cycle (12 h light at 30 °C/12 h dark at 20 °C), these mutants develop leaves of an almost normal green colour (Yoo *et al.*, 2009). In our present study, the *val1* mutant initially produced albino new leaves, but these subsequently turned green and only a small number of leaves showed marginal albinism at later developmental stages (Fig. 1). The results indicated that *val1* is a novel green-revertible albino mutant, for which the phenotype is regulated by development and is independent of temperature.

Nucleotide metabolism is a crucial process in all living organisms (Smith and Atkins, 2002). Although the *de novo* biosynthesis of purine nucleotides is essential for plant growth and development, little information is available on the molecular mechanism of the purine biosynthesis pathway in plants. The Arabidopsis gene *CIA1* encodes a Gln phosphoribosyl pyrophosphate amidotransferase 2 (ATase2), which is one of three ATase isozymes responsible for the first step of *de novo* purine biosynthesis. The *cia1* mutant displays normal green cotyledons, together with small and albino/pale-green mosaic leaves that contain slightly smaller cells but only half the cell number of the WT, which indicates that *de novo* purine biosynthesis is important for chloroplast biogenesis and cell division (Hung *et al.*, 2004). Our present study showed that *VAL1* encodes a phosphoribosylamine-glycine ligase, the second enzyme in the *de novo* purine biosynthesis pathway. In the *val1* mutant, chloroplast development was severely defective and chlorophyll metabolism was disrupted, which resulted in the dynamic green-revertible albino leaf phenotype. Taken together, the findings indicated that VAL1 is a crucial enzyme that regulates the *de novo* purine biosynthesis pathway, which may be involved in the development of leaf colour in rice. In addition, the *val1* mutant developed narrow leaves, caused by diminished cell division owing to the reduced content of cytokinins in the leaf (Fig. 7), and led to a reduced number of vascular bundles and fewer cells between the small vascular bundles (Fig. 1). Thus, the *de novo* purine biosynthesis pathway may also be involved in the process of cell division in rice. The location of the pathway within plant cells is presently still in dispute, although the subcellular localization of multiple enzymes provides convincing evidence (Smith and Atkins, 2002). Our current study showed that VAL1 contained a chloroplast transit peptide and was localized in the chloroplast (Fig. 3), which indicated that at least part of the *de novo* purine biosynthesis occurs in the chloroplasts in rice.

It is worth considering why the mutation of *VAL1* caused a dynamic green-revertible albino phenotype. One hypothesis is that VAL1 paralogs might perform the same or similar functions at later growth stages, and eventually offset the loss caused by mutation in the *VAL1* gene. This explanation was confirmed by phylogenetic analysis. There was one *VAL1*-like gene, *LOC_Os12g09540*, which showed high similarity to VAL1 at the amino acid level (Fig. 4; Supplementary Fig. S2), and *LOC_Os12g09540* was also highly expressed in leaves, with the highest levels detected in L3U (Fig. S3A, B). One reasonable interpretation is that there is a threshold level of purine nucleotides that needs to be met for normal chloroplast biogenesis and chlorophyll metabolism. Under rapid growth conditions, purine nucleotides are channelled into cell division, the purine nucleotide level in the mutant drops below the threshold, and chloroplasts fail to develop normally, causing the mutant to display albino leaves. When the division and growth rate of leaf cells are slower, purine nucleotides have time to accumulate in the mutant cells, eventually reaching the threshold level, and the mutant leaves subsequently turn green in the later stages. In *val1* seedlings, the contents of the *de novo* purine biosynthesis products adenine nucleotide (AMP) and guanine nucleotide (GMP) were lower than those of the WT seedlings (Fig. 5B), which indicated that the purine nucleotide level in the mutant dropped below the threshold. Furthermore, in the *val1* mutant, application of AMP, GMP, and AMP and GMP together partially restored the accumulation of chlorophyll in the *val1* mutant (Fig. 5D, E). Taken together, these results indicated that the dynamic green-revertible albino process may be regulated by the threshold level of purine nucleotides. In addition, purine nucleotides are maintained in intracellular pools through a combination of *de novo* synthesis and salvage pathways (Zrenner *et al.*, 2006). Previous studies have demonstrated that the strong demand for nucleotides in growing and dividing cells is met by *de novo* synthesis, whereas non-growing cells may be able to maintain their pools of nucleotides by salvaging (Ashihara and Crozier, 1999; Zrenner *et al.*, 2006). *VAL1* achieved the highest expression levels in the WT leaves at the L3 stage, and accumulated more in L3L but decreased dramatically in L3U (Fig. 3C). These observations suggested that VAL1 might play an important role at the early leaf development stage, with a relatively minor role in the mature leaf. Thus, another reasonable interpretation is that the *de novo* purine biosynthesis process regulates chloroplast development and chlorophyll metabolism at the early leaf development stage, and the salvage pathway regulates chloroplast development and chlorophyll metabolism in mature leaves.

## Supplementary data

Supplementary data are available at *JXB* online.

Fig. S1. The *val1* mutant is a temperature-insensitive green-revertible albino mutant.

Fig. S2. Protein sequence alignment of VAL1.

Fig. S3. Expression patterns of *LOC_Os12g09540*.

Fig. S4. VAL1 participates in the *de novo* purine biosynthesis pathway.

Fig. S5. Exogenous application of 6-BA to the wild-type and *val1* mutant.

Fig. S6. Expression of genes associated with chloroplast development, photosynthesis, and pigment metabolism in the wild-type and *val1* mutant in the heading stage.

Table S1. Primers used in the study.

Table S2. DEGs annotated within chloroplast development and photosynthesis.

Table S3. DEGs annotated within pigment biosynthesis.

## Supplementary Material

Supplementary Figures S1-S6 and Tables S1-S3
