# Supplementary material for: VIRESCENT-ALBINO LEAF 1 regulates leaf colour development and cell division in rice
Source: J Exp Bot. 2018 Aug 8;69(20):4791–804. doi: 10.1093/jxb/ery250 (PMC6137968; doi:10.1093/jxb/ery250)
Supplement: Supplementary Figures S1-S6 and Tables S1-S3 [file ery250_suppl_supplementary_figs-s1-s6_tables-s1-s3.pdf]

Supplementary Figures

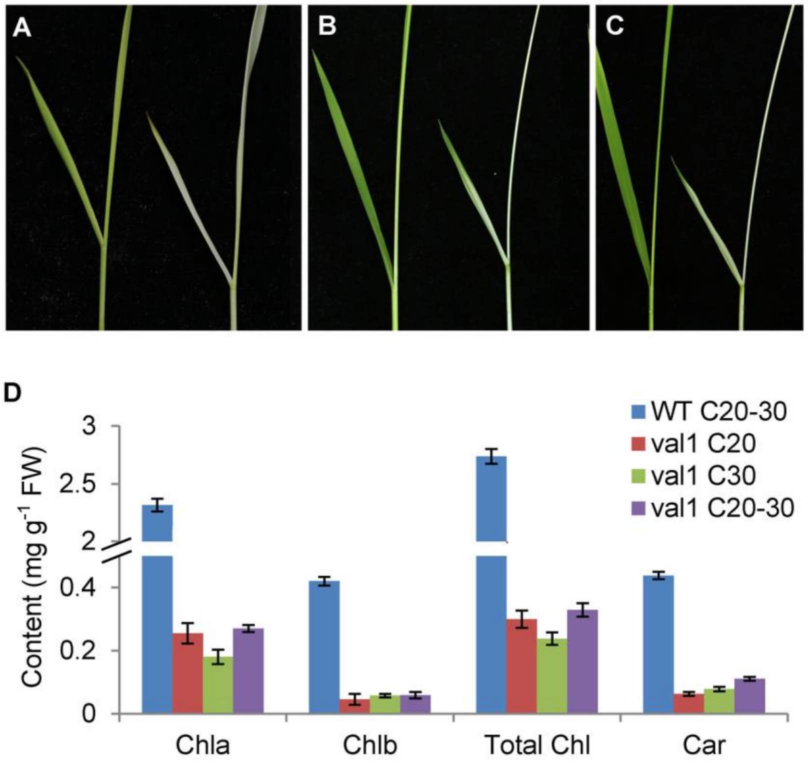

Fig. S1. The *val1* mutant is a temperature-insensitive green-revertible albino mutant. (A–C) WT (left) and *val1* (right) seedlings grown under 20 ℃ (C20) (A), 30 ℃ (C30) (B), and alternate light/dark cycle (12 h light at 30 ℃/12 h dark at 20 ℃ [L30 ℃/D20 ℃]) (C). (D) Chlorophyll content of the leaf of the WT and *val1* in (A–C).

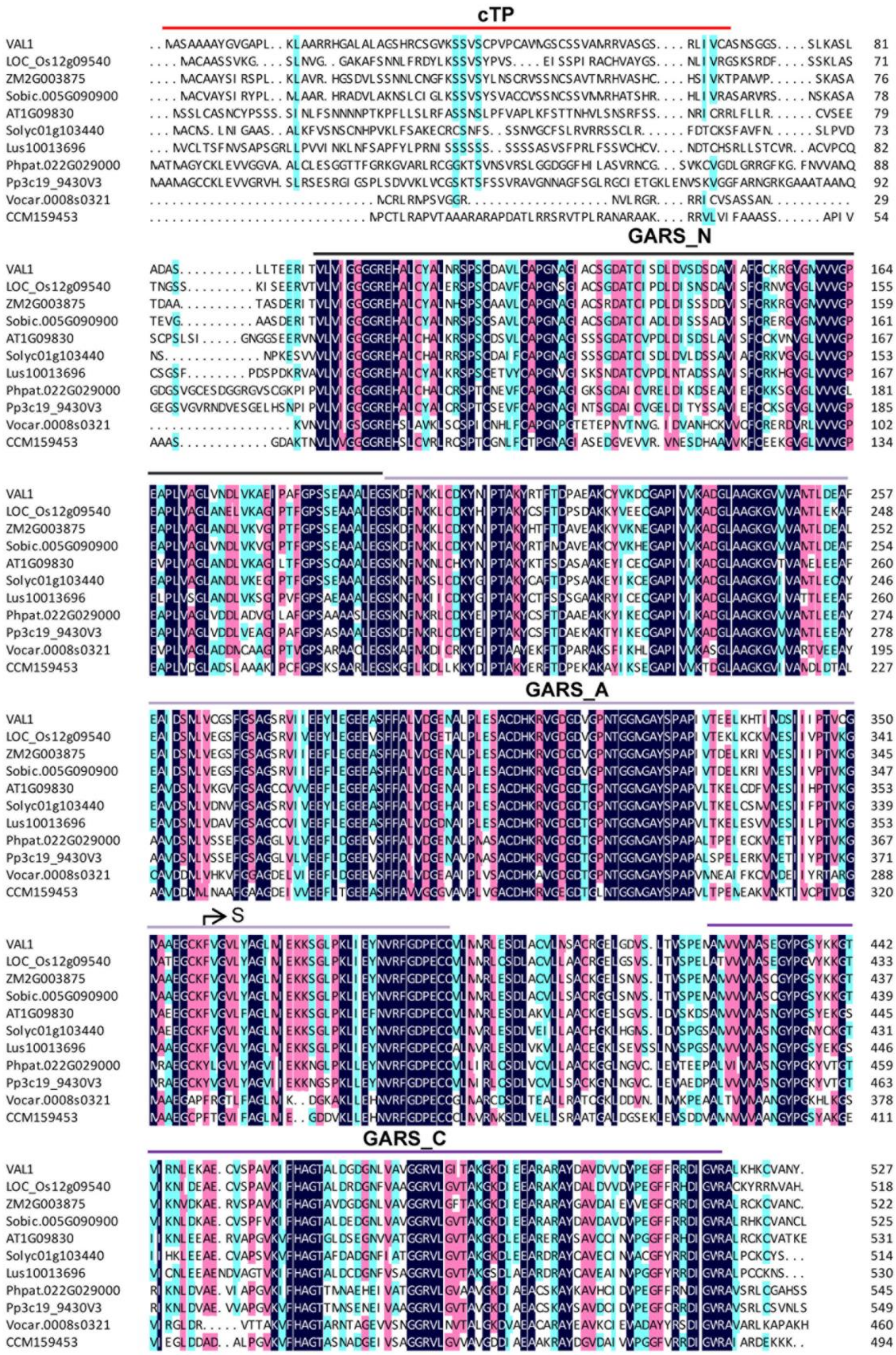

Fig. S2. Protein sequence alignment of VAL1. VAL1 is composed of a cTP (residues 1–68) and three conserved domains, named GARS_N, GARS_A, and GRAS_C, with lines in different colours above the sequence. The black arrow indicates the mutation site in *val1*. cTP, chloroplast transit peptide.

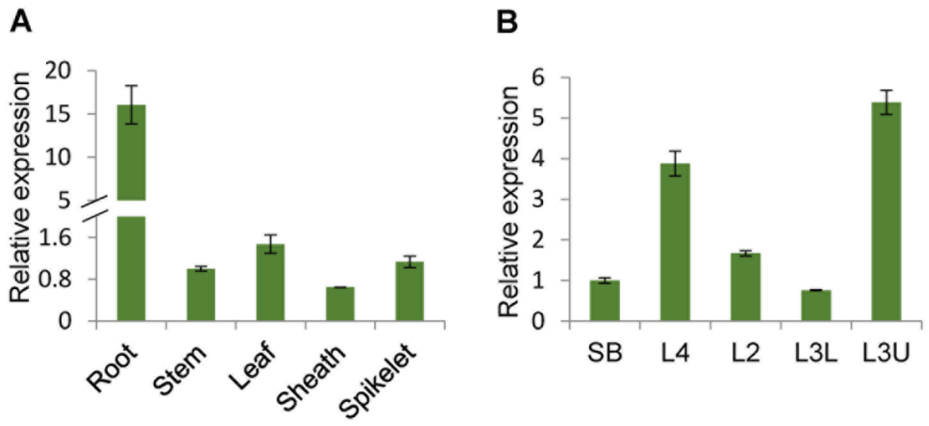

Fig. S3. Expression pattern of *LOC_Os12g09540*. (A) Expression pattern of *LOC_Os12g09540* in different tissues as indicated by real-time PCR. (B) Expression pattern of *LOC_Os12g09540* in the leaf as indicated by real-time PCR. SB, shoot base; L4, fourth leaf; L2, second leaf; L3L, basal half of the third leaf; L3U, upper half of the third leaf. Error bars represent the standard deviation (SD) of three biological repeats.

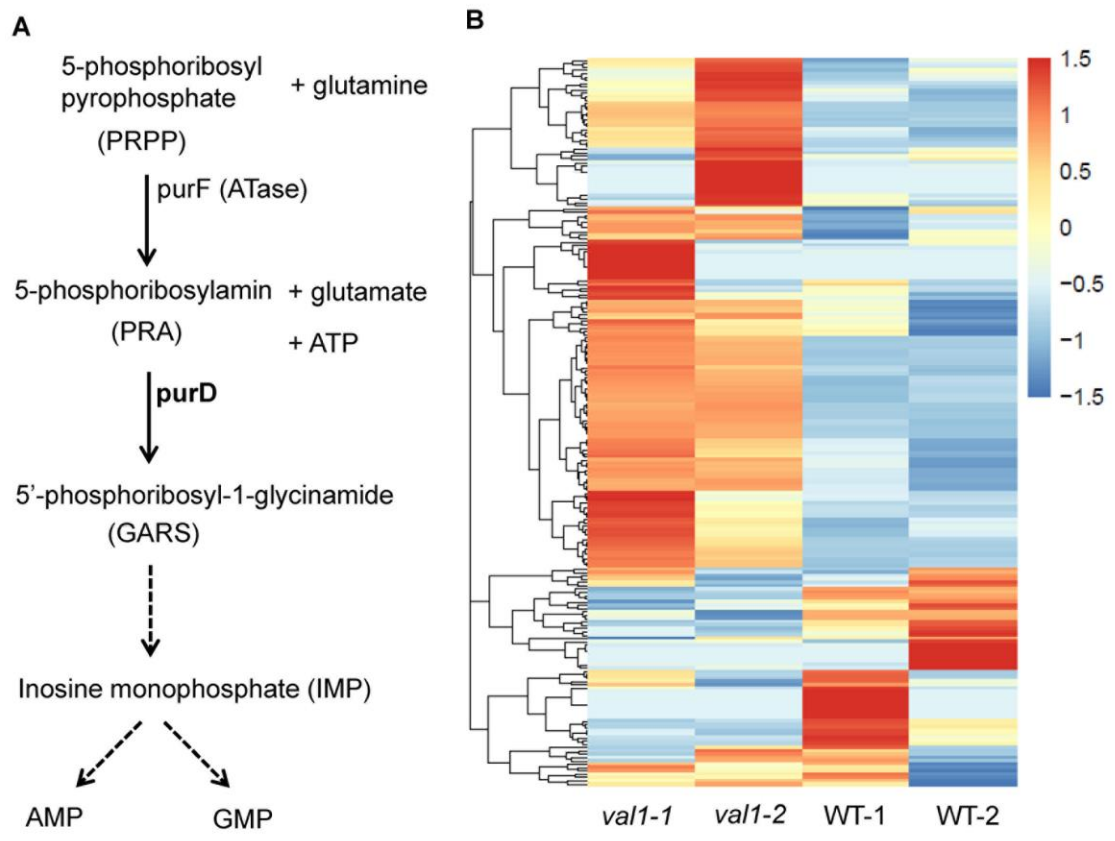

Fig. S4. *VAL1* participates in the *de novo* purine biosynthesis pathway. (A) The *de novo* purine biosynthesis process. (B) Heat map of differentially expressed genes associated with the *de novo* purine biosynthesis pathway.

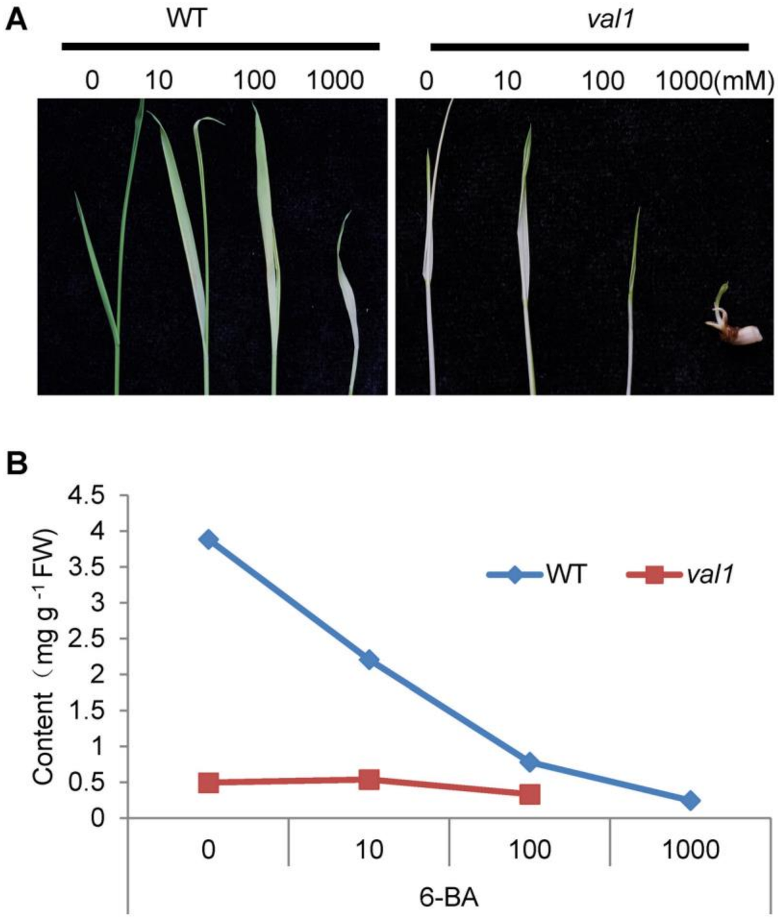

Fig. S5. Exogenous application of 6-BA to the wild type (WT) and *val1* mutant. (A) Leaf morphology of the WT and *val1* mutant after growth in Murashige and Skoog liquid medium supplemented with various concentrations of 6-BA for 2 weeks. (B) Chlorophyll content of the plants of the WT and *val1* shown in A. FW, fresh weight. Error bars represent the standard deviation (SD) of three biological repeats.

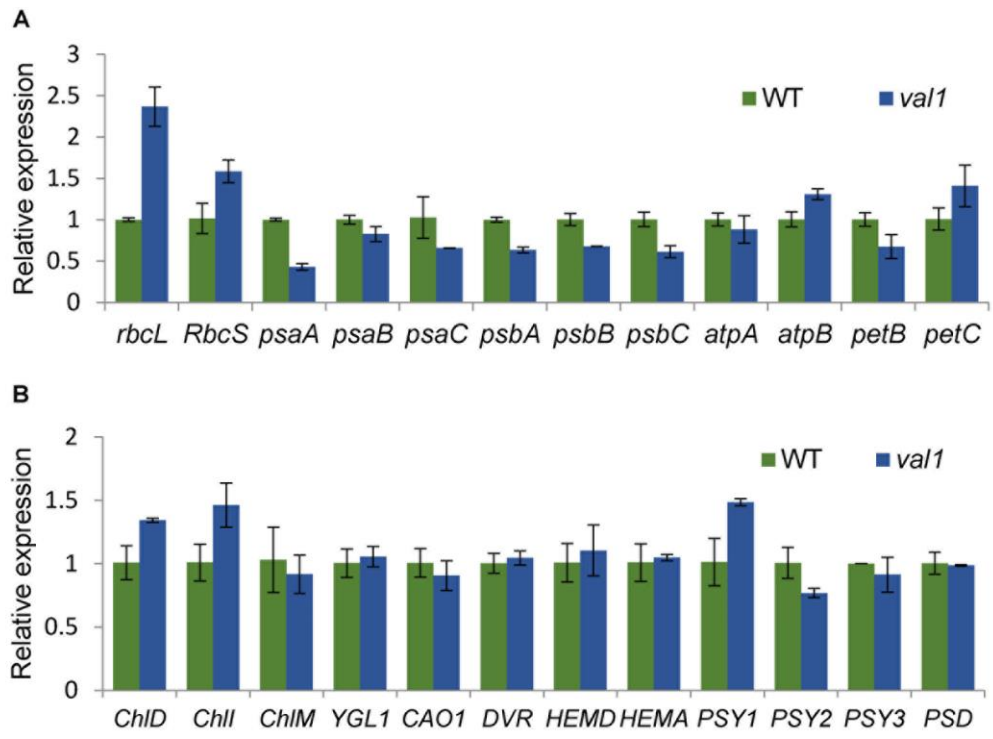

Fig. S6. Expression of genes associated with chloroplast development, photosynthesis, and pigment metabolism in the wild type (WT) and *val1* mutant in heading stage. (A) Expression analysis of genes associated with chloroplast development and photosynthesis in the WT and *val1*. (B) Expression analysis of genes associated with pigment metabolism in the WT and *val1*. Error bars represent the standard deviation (SD) of three biological repeats.

Supplemental Table 1. Primers used in the study.

Purpose	Primer name	Sequence	Remarks
Mapping	SSR8-1F	ATTGCTAAAGATGATTTGGAACTA	SSR
	SSR8-1R	GGGACCTAGAAACATCATCTCC	SSR
	ID30F	CGCCAGCAATGTAGGTTTAT	Indel
	ID30R	CATGCTTGCTAAACAGATACAGAC	Indel
Complementary	VAL1C-1F	GCCgaattcGTTGGGTTCAAATCCCACCTTTCT	EcoRI
	VAL1C-1R	CTCTCATTGGAGCAAATGTTCATTGTC	KpnI
	VAL1C-2F	CTCAGAAGCTGCGGCTTTAGAAGGAT	KpnI
	VAL1C-2R	GCCggatccCTCGGCCCAGTTAAGGCCAGCT	BamHI
RNA interference	VAL1Ri-F1	gccggatccGAATCCGATCTAGCACAGGTTCTGAT	BamHI
	VAL1Ri-R1	gccggtaccGCCAGTCAACAACATCTACTGCATCAT	KpnI
	VAL1Ri-F2	gccgagctcGAATCCGATCTAGCACAGGTTCTGAT	SacI
	VAL1Ri-R2	gccactagtGCCAGTCAACAACATCTACTGCATCAT	SpeI
Over-expression	VAL1OE-F	gccggtaccATGGCGTCTGCTGCTGCCGCT	KpnI
	VAL1OE-R	gccactagtGTAATTGGCCACTTGCTTGTGCTTCAGTG	SpeI
Subcellular localization	VAL1pAN-F	gccactagtATGGCGTCTGCTGCTGCCGCT	SpeI
	VAL1pAN-R	gccggatccGTAATTGGCCACTTGCTTGTGCTTCAGTG	BamHI
	Actin-F	TGCTATGTACGTCGCCATCCAG	
	Actin-R	AATGAGTAACCACGCTCCGTCA	
	qVAL1-F	CCTGCACCAATAGTGACAGAAGAGCT	
	qVAL1-R	CGCATCATAAGAACCTGGCATTCT	
	qVAL2-F	TGGAGAAACTGCTTTGCCTCTTGAAT	
	qVAL2-F	CATACAACACACCGACGAACTTGCAT	
	qrbcL-F	GGAGGGACGTATGTCACCACAAAC	
	qrbcL-R	GAGTTACTCGGAATGCTGCCAAG	
	qRbcS-F	GTGGCAACTAAGCCGTCATCGTC	
	qRbcS-R	TGCCTCACCCAACAACATATAGTCGT	
	qpsaA-F	TGGGGTTGATCCTAAGGAGATACCA	
	qpsaA-R	CCTCCGCGAAAACTAAGAAATTCTG	
	qpetA-F	GCAGCAAGGTTATGAAAACCCAC	
	qpetA-R	AACAGCACCCACATTCAACCCT	
	qpetG-F	TCCCCTCGAACAAATCGAAATG	
	qpetG-R	CAACTGATCCCCACGTCTGTATT	
	qatpA-F	TGAATCTCCTGCTCCGGGTATAAT	
	qatpA-R	TGCTGTTTTGCCGGTTTGTCT	
	qLhcpII-F	GAAGAAGATCAAGAACGGCC	
	qLhcpII-R	TTGCCGGGGACGAAGTTGGT	
	qpsaB-F	TGGCAGGGCAACGTTTCACAATT	
	qpsaB-R	CAGTCGCCCAAACAAGATGTCCAAAT	
	qpsaC-F	GTGTACGAGCTTGTCCAACAGATGTAT	
	qpsaC-R	GCAGGCGGATTCGCATCTCTTAC	
	qpsbA-F	GCGGTTCCCTATTCAGTGCTATG	

	qpsbA-R	TAACCATGAGCGGCCACAATATT
	qpsbB-F	TAGTTTCTGGTTGGGCTGGCTC
	qpsbB-R	CTCCAACCACCCCACGAATTG
	qpsbC-F	GTTCCCCAACGGGAGAGGTTAT
	qpsbC-R	GAGCCTAAAGGAGCATGGGTCAT
	qatpB-F	TCGCAATTCTTGGGTTGGATGA
	qatpB-R	CAACATACTTTCCCGGAGAACCG
	qpetB-F	TTCAGACCTCGCAACCAGACTG
	qpetB-R	AACAAAAGGCAAGGGTTCTTCGA
	qpetC-F	CTCAAGGGTGACCCGACGTACCT
	qpetC-R	AGGGGCAGATGAACTTGTTCTCG
	qChlD-F	GCTTGCAGAAAGCTACACAAGC
	qChlD-R	AGGCCGTGAGCTAAAGGAGA
	qChlI-F	GTTCGAGCCTGGTTTGCTTGC
	qChlI-R	CTCTCCACGGTGTTCCATCCTG
	qChlM-F	CCATCCATTGGTCTCCTTATGACA
	qChlM-R	GTAGCCTACTTACCATCAATGAGTC
	qYGL1-F	GATAGAGCTCTGGGGCTTCAGTC
	qYGL1-R	GCTTGCCGGAACTGAAAAGGTAG
qRT-PCR	qCAO1-F	GACACCTTCATCTGGGCTTCAA
	qCAO1-R	CGAGAGACATCCGGTAGAGC
	qDVR-F	CAGGTCGAGACCGTCAAGAAC
	qDVR-R	ATGACCTGGATCGGCACCTTG
	qHEMD-F	TGGAAGGCTGCTGGAAATCCTAAG
	qHEMD-R	TCCTTGGAAGCTCTGAGGCCAA
	qHEMA-F	GAACTCACCAGTCTGAATCATATTGA
	qHEMA-R	CATCCAGTCTACCACTTCTCTAATCC
	qPSY1-F	GCCTCAAGCAGGCCTATCATC
	qPSY1-R	GTGATGTGCGAGGCATTTGGTC
	qPSY2-F	GACAAATTCTGCGTGCCAGGTT
	qPSY2-R	GACAGCAGCTTCTTTGCCTTGTT
	qPSY3-F	TTCAGACAGGCCGAAGAAGGC
	qPSY3-R	GTAGGCCCTCTTGGTGAAGTTGT
	qPDS-F	GTTCCTGATCGAGTGAACGATGAAG
	qPDS-R	CGAACATGGTCAACAATAGGCATG
	VAL1-F	CGATGCCGTTATCGCGTTCT
In situ	VAL1SP6-R	agatttaggtgacactatagCTTCAAATGCCTCATCCAAAGTC
	His4-F	ATGTCGGGCCGCGGCAAG
	His4SP6-R	agatttaggtgacactatagAATCAGCCGCCGAAGCCGTAG

Supplemental Table 2. DEGs annotated within the chloroplast development and photosynthesis.

gene_name	gene_name	*val.1*	*val.2*	WT.1	WT.2	log2FoldChange
psbR	OsR498G0713510500.01	82.49	73.38	433.84	460.44	-2.62
psaG/K	OsR498G0917697300.01	94.86	116.48	409.78	405.81	-2.05
psbK	OsR498G1019218800.01	5.73	5.04	14.34	17.54	-1.66
psbD	OsR498G0917274300.01	7.81	7.79	17.16	21.16	-1.40
psaD	OsR498G0816613600.01	956.77	567.22	1647.92	1682.06	-1.36
rbcL	OsR498G1120276900.01	0.81	0.63	1.63	1.46	-1.20
psaA/B	OsR498G1120159700.01	1.24	1.40	2.55	2.97	-1.16
psbY	OsR498G0815087200.01	285.10	304.07	544.03	614.73	-1.16
psbZ	OsR498G0611904200.01	20.98	16.52	32.54	45.43	-1.14
psaI	OsR498G1221413300.01	626.93	581.55	1153.06	1208.58	-1.08
psaL	OsR498G1221413300.01	626.93	581.55	1153.06	1208.58	-1.08
psaO	OsR498G0408537300.01	605.27	616.48	1145.63	1206.79	-1.06
psbC	OsR498G0510353500.01	32.20	24.33	49.99	58.18	-1.04
psaE	OsR498G0714154900.01	596.77	582.00	1046.45	1085.33	-0.97
psaN	OsR498G1221059300.01	455.84	454.94	793.96	840.49	-0.95
psbP	OsR498G0713493200.01	621.69	646.51	1107.05	1160.35	-0.95
psbQ	OsR498G0714524300.01	565.81	523.64	872.61	1046.53	-0.93
RBCS	OsR498G1221348500.01	1749.74	1680.83	2875.74	3119.97	-0.92
psbW	OsR498G0102077400.01	916.04	987.79	1582.83	1658.34	-0.88
psaF	OsR498G0307145600.01	813.76	822.40	1368.49	1418.29	-0.88
psbB	OsR498G0022235700.01	6.95	6.65	10.76	12.02	-0.85
petB	OsR498G0612784100.01	9.43	8.64	13.29	16.81	-0.84
psbO	OsR498G0101060700.01	1334.45	1218.84	1907.07	2337.54	-0.84
psaH	OsR498G0511332700.01	797.45	815.53	1312.03	1347.75	-0.83
psbM	OsR498G0612784300.01	11.35	9.34	15.22	18.95	-0.83
psaC	OsR498G0917714700.01	0.06	0.00	0.15	0.06	-0.78
psbX	OsR498G0305924900.01	812.53	847.77	1280.99	1345.54	-0.77
psbE	OsR498G0714574700.01	0.13	0.14	0.15	0.20	-0.42
psb27	OsR498G0305888900.01	233.47	246.19	295.66	290.50	-0.40
petC	OsR498G0714559100.01	478.14	498.17	571.35	560.00	-0.32
psb29	OsR498G0714568200.01	162.91	161.45	159.42	169.76	-0.14
petM	OsR498G0307104900.01	311.35	340.25	320.08	313.58	-0.07
psbA	OsR498G1019134500.01	0.68	0.49	0.41	0.40	0.42
psaJ	OsR498G0203411700.01	0.04	0.01	0.03	0.02	0.44

Supplemental Table 3. DEGs annotated within the pigment biosynthesis.

gene_name	gene_name	*val.1*	*val.2*	WT.1	WT.2	log2FoldChange
PORA	OsR498G0409495300.01	294.35	307.80	2118.71	2144.09	-2.93
RCCR1	OsR498G1018622600.01	5.11	6.47	13.26	10.94	-1.16
CAO1	OsR498G1019221000.01	80.00	77.73	148.60	151.97	-1.05
PORB	OsR498G1018982300.01	169.31	176.19	285.80	300.16	-0.87
YGL8	OsR498G0100668500.01	301.97	287.45	468.58	530.71	-0.87
PSY1	OsR498G0613340000.01	40.80	41.30	59.99	65.62	-0.73
PPOX1	OsR498G0100695700.01	37.40	40.57	40.52	41.61	-0.48
HEMA	OsR498G1019000200.01	111.46	130.62	149.97	161.88	-0.48
YGL2	OsR498G0612895800.01	49.76	46.25	56.42	61.35	-0.41
HEME	OsR498G0612895800.01	49.76	46.25	56.42	61.35	-0.41
ChlH	OsR498G0305849500.01	114.52	108.61	123.77	136.42	-0.34
HEMH	OsR498G0510654700.01	33.89	33.46	37.09	33.84	-0.19
ChlI	OsR498G0306382200.01	91.36	78.31	86.25	91.99	-0.18
DVR	OsR498G0305948400.01	23.38	24.19	23.50	24.40	-0.12
ChlM	OsR498G0611575400.01	76.99	81.38	75.87	78.03	-0.07
HEMB	OsR498G1221906500.01	3.37	4.27	3.45	3.69	-0.01
ZEBRA2	OsR498G1120394100.01	8.69	7.64	7.75	7.07	0.04
HEMD	OsR498G0305349500.01	22.08	15.05	12.23	20.28	0.07
ChlD	OsR498G0307254700.01	38.12	35.34	28.93	29.59	0.21
PDS	OsR498G0305342900.01	40.61	30.75	31.40	24.88	0.23
ChlG	OsR498G0510598100.01	45.23	44.03	33.75	36.52	0.23
YGL1	OsR498G0510598100.01	45.23	44.03	33.75	36.52	0.23
HEMC	OsR498G0203046700.01	155.18	157.62	105.57	104.35	0.46
CAO2	OsR498G1019220400.01	2.41	2.15	1.21	1.19	0.81
HEML	OsR498G0816520200.01	131.38	126.99	20.33	98.44	0.84
